# Fibrin and Marine-Derived Agaroses for the Generation of Human Bioartificial Tissues: An Ex Vivo and In Vivo Study

**DOI:** 10.3390/md21030187

**Published:** 2023-03-17

**Authors:** Olimpia Ortiz-Arrabal, Ainhoa Irastorza-Lorenzo, Fernando Campos, Miguel Ángel Martín-Piedra, Víctor Carriel, Ingrid Garzón, Paula Ávila-Fernández, María José de Frutos, Emilio Esteban, Javier Fernández, Agustín Janer, Antonio Campos, Jesús Chato-Astrain, Miguel Alaminos

**Affiliations:** 1 Tissue Engineering Group, Department of Histology, University of Granada and Instituto de Investigación Biosanitaria ibs.GRANADA, E18016 Granada, Spain; 2 Doctoral Program in Biochemistry and Molecular Biology, University of Granada, E18016 Granada, Spain; 3 Hispanagar, SA, E09001 Burgos, Spain

**Keywords:** tissue engineering, biomaterials, fibrin, agarose, biocompatibility

## Abstract

Development of an ideal biomaterial for clinical use is one of the main objectives of current research in tissue engineering. Marine-origin polysaccharides, in particular agaroses, have been widely explored as scaffolds for tissue engineering. We previously developed a biomaterial based on a combination of agarose with fibrin, that was successfully translated to clinical practice. However, in search of novel biomaterials with improved physical and biological properties, we have now generated new fibrin-agarose (FA) biomaterials using 5 different types of agaroses at 4 different concentrations. First, we evaluated the cytotoxic effects and the biomechanical properties of these biomaterials. Then, each bioartificial tissue was grafted in vivo and histological, histochemical and immunohistochemical analyses were performed after 30 days. Ex vivo evaluation showed high biocompatibility and differences in their biomechanical properties. In vivo, FA tissues were biocompatible at the systemic and local levels, and histological analyses showed that biointegration was associated to a pro-regenerative process with M2-type CD206-positive macrophages. These results confirm the biocompatibility of FA biomaterials and support their clinical use for the generation of human tissues by tissue engineering, with the possibility of selecting specific agarose types and concentrations for applications requiring precise biomechanical properties and in vivo reabsorption times.

## 1. Introduction

Several types of biomaterials have been used for the generation of bioartificial tissues by tissue engineering [1,2,3,4]. In general, biomaterials used in tissue engineering should be biocompatible, biodegradable and non-immunogenic, and should support cell attachment, growth and function [5,6]. In addition, biodegradation of the biomaterials grafted in vivo should be associated with the synthesis of native extracellular matrix (ECM), allowing tissue regeneration [5], and it has been suggested that the specific biodegradation properties and kinetics of biomaterials used in tissue engineering should be modulated to each specific application [7].

One of the most important properties of biomaterials used in tissue regeneration are their biomechanical properties [8,9]. In fact, the stiffness and elasticity of biomaterials can significantly affect the regenerative potential of bioartificial tissues and influence cell viability and cell function [10]. Therefore, controlling their biomechanical properties is a requirement of biomaterials used in tissue regeneration, and each specific application should take into account the distinct biomechanical properties of each specific material [7]. 

Among the most promising natural biomaterials, fibrin has been extensively used in tissue engineering due to its unique accessibility and biological properties [11]. Fibrin is a fibrillar protein involved in blood clotting, wound healing and inflammation, among other relevant functions [12]. In tissue engineering, fibrin can form highly biocompatible, bioactive three-dimensional porous matrices able to promote cell function, growth and differentiation [13]. In addition, this biomaterial can be easily obtained from the blood of a patient, thus eliminating the risk of immune rejection and reducing manufacturing costs [14]. As scaffold, fibrin has been used in tissue engineering of numerous tissues and organs, including bone, cartilage, skin and cardiac tissue, among others [13,15,16,17,18]. However, the mechanical properties of fibrin hydrogels, along with the rapid degradation rate of fibrin grafted in vivo, make it necessary to improve the intrinsic properties of this biomaterial in order to overcome these limitations [19].

Marine polysaccharides are other natural biomaterials that has been used in tissue engineering of different tissues and organs [20]. The inherent natural properties of these marine products such as biodegradability and biocompatibility, make agarose a perfect product to be used a scaffold in tissue engineering [21,22,23]. Agarose is a natural polysaccharide that exhibits controlled self-gelling properties, water solubility, and high biocompatibility, and it has been demonstrated that this material could enhance cell proliferation and activity [24]. Several types of agaroses have been described so far, and we recently demonstrated that the concentration and the specific type of agarose used in tissue engineering can significantly influence the biomaterial biomechanical properties, biological functions, and in vivo biocompatibility [23]. Therefore, specific concentrations and types of agarose should be used for each specific application [23]. Although agarose has adequate biological and biomechanical properties, this product has several limitations, especially regarding cell differentiation [22]. For this reason, agarose biomaterials are normally combined with other biomaterials showing more adequate cell properties, such as chitosan and graphene oxide, silk fibroin, collagen and fibrin [22,25,26].

Fibrin-agarose (FA) biomaterials were first described by our group for the generation of cornea substitutes with promising results [27]. Subsequently, FA scaffolds were applied to the generation of numerous tissues and organs, such as the skin, nerve, tendon, sclera and oral mucosa [28,29,30,31,32]. Previous studies showed that the combination of these two materials resulted in a significant improvement of the biomaterials’ biomechanical properties as compared to fibrin hydrogels, especially when nanostructuration is applied [33,34]. Moreover, the biocompatibility and the in vivo biosafety of the constructs have been also demonstrated [35], which allowed us to use this biomaterial in patients [36,37]. Although the biological and biomechanical properties of FA scaffolds are adequate, the possibility of enhancing these properties by combining fibrin with different types of agaroses has not been assessed to date.

## 2. Results

### 2.1. Cell Viability of Human Fibroblasts Immersed within Fibrin-Agarose Biomaterials

**Figure 1:**
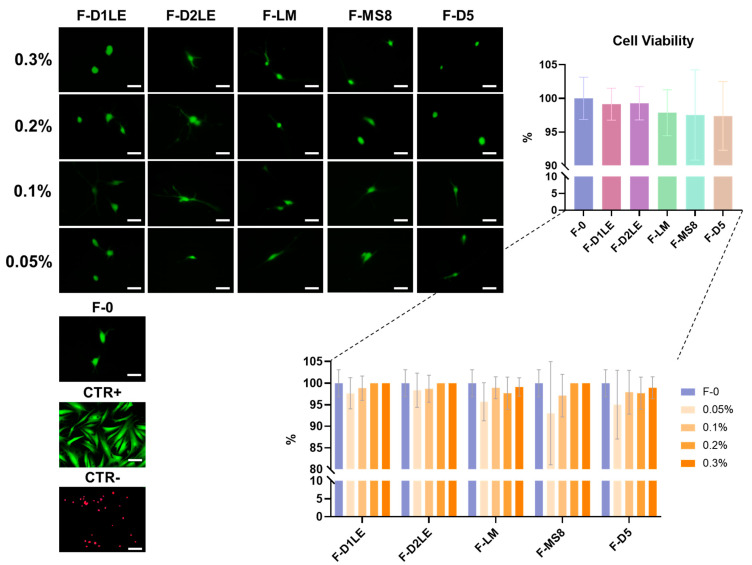
Cell viability assessment of human cells immersed in the different bioartificial tissues generated in this work. Live/Dead images show live cells in green and dead cells in red. The percentage of live cells was calculated in each sample and represented in each global group of samples with specific agarose typess and in each specific sample, with the different concentrations of agarose (0%, 0.05%, 0.1%, 0.2% and 0.3%). F-0: fibrin biomaterials without agarose; F-D1LE: fibrin-agarose biomaterials containing D1LE agarose; F-D2LE: fibrin-agarose biomaterials containing D2LE agarose; F-LM: fibrin-agarose biomaterials containing LM agarose; F-D5: fibrin-agarose biomaterials containing D5 agarose. No significant differences were found among the different groups generated in this study. Scale bar = 100 µm.

Analysis of human cells immersed within the different fibrin and FA biomaterials generated in the present work revealed that all biomaterials were highly biocompatible *ex vivo*, as shown in Figure 1 and Supplementary Tables S1 and S2. Specifically, our results showed that more than the 95% of fibroblasts within the hydrogel were alive in all study groups (average 98.24 ± 4.35% of live cells for all FA biomaterials considered together). Differences between the F-0 group and the rest of the study groups (biomaterials containing the different agarose types and concentrations) were not statistically significant (*p* > 0.05), as was the case of all other comparisons among specific types of samples (*p* > 0.05) (Supplementary Table S2).

### 2.2. Biomechanical Properties of Fibrin-Agarose Biomaterials

**Figure 2:**
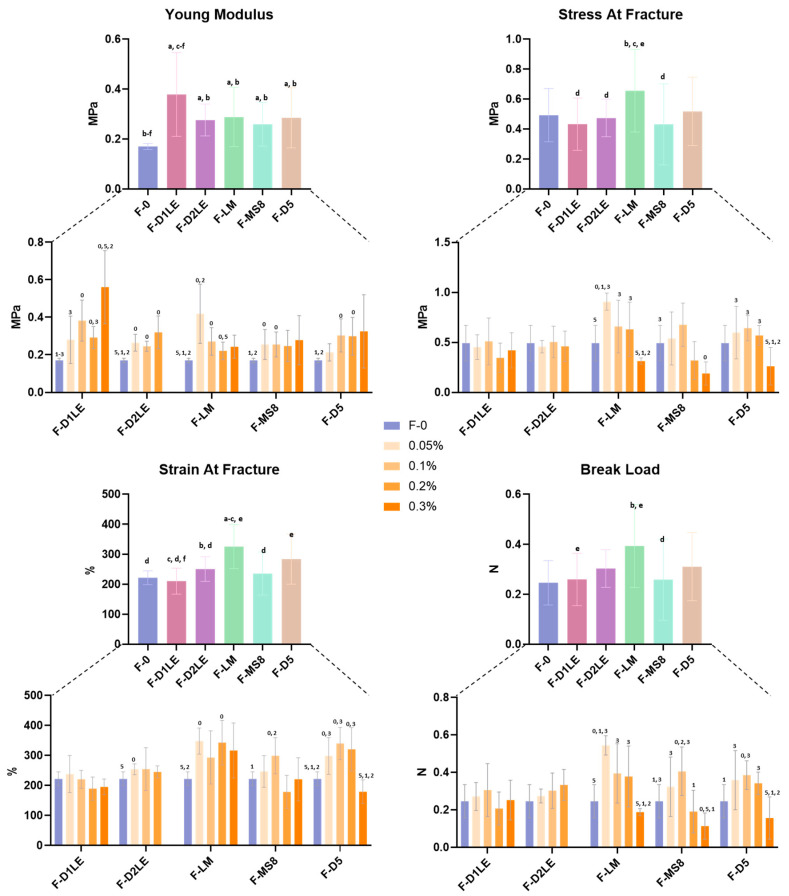
Biomechanical behavior of the different bioartificial tissues generated in this work. Results of each global group of samples regardless of the agarose concentration and each specific type of sample, with the different concentrations of agarose (0%, 0.05%, 0.1%, 0.2% and 0.3%) are represented. F-0: fibrin biomaterials without agarose; F-D1LE: fibrin-agarose biomaterials containing D1LE agarose; F-D2LE: fibrin-agarose biomaterials containing D2LE agarose; F-LM: fibrin-agarose biomaterials containing LM agarose; F-D5: fibrin-agarose biomaterials containing D5 agarose. In the global samples, statistically significant differences are labeled as “a”, “b”, “c”, “d”, “e” and “f” for significant differences with F-0, F-D1LE, F-D2LE, F-LM, F-MS8 and F-D5, respectively. In the lower graphs, statistically significant differences among concentrations are labeled with 0 (differences with the F-0 group), 5 (differences with the 0.05% group), 1 (differences with the 0.1% group), 2 (differences with the 0.2% group), and 3 (differences with the 0.3% group).

All FA biomaterials generated in the present work were able to form a homogeneous solid hydrogel, except for FA containing D2LE at the concentration of 0.3%, which tended to jellify very rapidly and could not be properly combined with fibrin to form a homogeneous gel supporting the analysis and was therefore not measured. For the rest of the samples, assessment of the main biomechanical parameters in FA biomaterials showed that the type and concentration of agarose influenced the biomechanical properties of the biomaterials analyzed in this work (Figure 2 and Supplementary Tables S1 and S2). For the Young’s modulus, results corresponding to all global groups of FA samples were significantly higher than the F-0 group devoid of agarose, with differences being statistically significant for all types of agarose (see *p* values in Supplementary Table S2). Additionally, the comparison of global groups of FA samples revealed that F-D1LE had a significantly higher Young’s modulus than F-D2LE, F-LM, F-MS8, and F-D5 global groups. However, there were no significant differences among F-D2LE, F-LM, F-MS8, and F-D5 global groups (*p* > 0.05) (Figure 2 and Supplementary Tables S1 and S2). When specific types of samples were compared, we found that the Young’s modulus was significantly higher in most FA biomaterials as compared to F-0 samples, and that some differences were found among specific types of samples that are summarized in Figure 2, and Supplementary Table S2. 

For the stress at fracture, our study found a significant increase of this parameter in the global group of F-LM biomaterials as compared to F-D1LE, F-D2LE and F-MS8, with the highest stress at fracture values found in 0.05% F-LM specific samples. Similarly, F-LM showed the highest strain at fracture, with significant differences with F-0, F-D1LE, F-D2LE and F-MS8 global groups. Finally, the break load parameter revealed significant differences between F-LM and F-D1LE and F-MS8 global groups. For both the strain at fracture and the break load, specific differences summarized in Figure 2, and Supplementary Tables S1 and S2 suggest that the highest values correspond to 0.05% concentrations of LM agarose in the FA samples.

### 2.3. Systemic Effects of Each Type of Biomaterial Grafted In Vivo in Laboratory Rats

**Figure 3:**
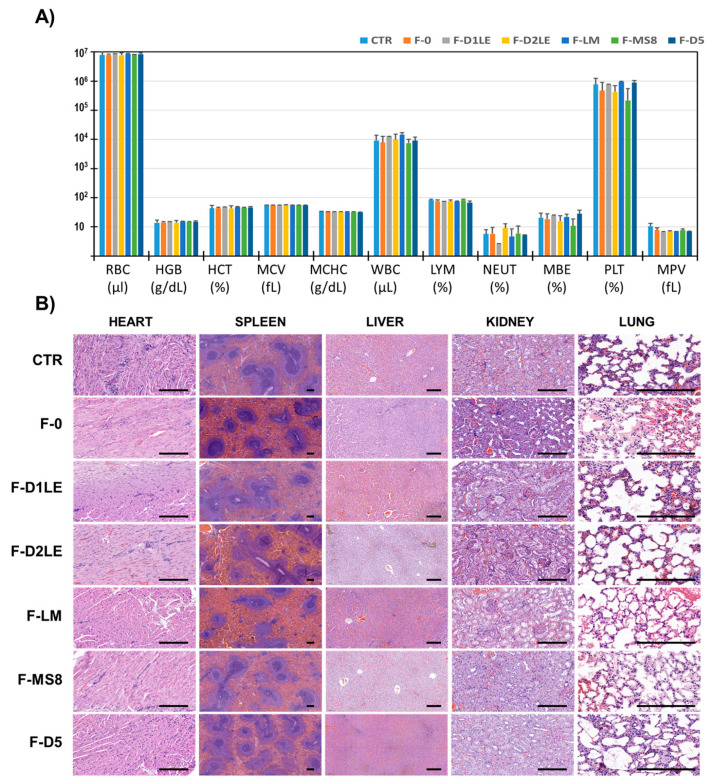
In vivo biocompatibility analysis of the different biomaterials grafted in laboratory animals for 30 days. (**A**) Analysis of hematological parameters in peripheral blood. For each group of animals, average values of red blood cells (RBC), hemoglobin (HGB), hematocrit (HCT), mean cell volume (MCV), mean red blood cell hemoglobin content (MCHC), white blood cells (WBC), percentage of lymphocytes (LYM), percentage of neutrophils (NEU), percentage of monocytes–basophils–eosinophils (MBE), percentage of platelets (PLT) and mean platelet volume (MPV) are shown. (**B**) Histological analysis of five vital organs using hematoxylin-eosin staining (HE). F-0: fibrin biomaterials without agarose; F-D1LE: fibrin-agarose biomaterials containing D1LE agarose; F-D2LE: fibrin-agarose biomaterials containing D2LE agarose; F-LM: fibrin-agarose biomaterials containing LM agarose; F-D5: fibrin-agarose biomaterials containing D5 agarose. Scale bar: 200 µm.

When we analyzed the in vivo systemic effects of each type of biomaterial on laboratory animals, we first found that in vivo grafting of the different biomaterials was not associated with any alterations of the hematological parameters analyzed in the present study (Figure 3A). Differences with CTR animals were not statistically significant (*p* > 0.05) for any of the study groups (F-0, F-D1LE, F-D2LE, F-LM, F-MS8 and F-D5). 

Then, our histological analysis of five vital organs in each group of animals showed no differences among groups, and none of the animals showed any histological alterations of the analyzed organs. Specifically, the histological structure of the heart, spleen, liver, kidney and lung of CTR animals was similar to that of the animals grafted with the different biomaterials analyzed in the present study (F-0, F-D1LE, F-D2LE, F-LM, F-MS8 and F-D5). No signs of inflammation, necrosis, fibrosis, or malignant transformation were detected in any of the studied groups (Figure 3B).

### 2.4. In Situ Morphological Analysis of Biomaterials Grafted In Vivo in Laboratory Rats

**Figure 4:**
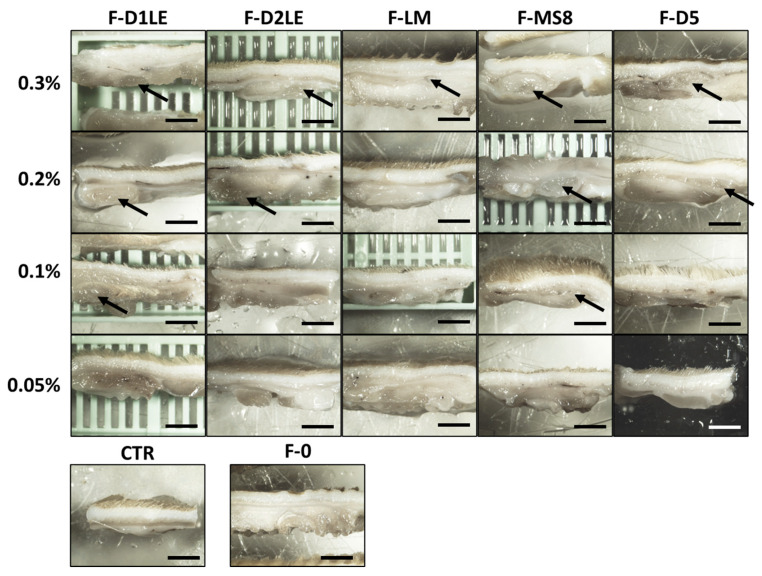
Macroscopical analysis of the implant sites in laboratory animals after 30 days of follow-up. Different types of FA biomaterials with different concentrations of agarose (0.05%, 0.1%, 0.2% and 0.3%) and fibrin biomaterials without agarose (F-0) were grafted in rats and photographed after 30 days of follow-up. Animals with no grafted material were included as CTR group. F-D1LE: fibrin-agarose biomaterials containing D1LE agarose; F-D2LE: fibrin-agarose biomaterials containing D2LE agarose; F-LM: fibrin-agarose biomaterials containing LM agarose; F-D5: fibrin-agarose biomaterials containing D5 agarose. Macroscopical biomaterial remains of the subcutaneous implants are labeled with arrows. Scale bar: 4 mm.

Macroscopical analysis of the samples grafted in vivo after the follow-up period revealed that all biomaterials became integrated with the host tissue, with no detectable local alterations. As shown in Figure 4, we found that the FA biomaterials with the highest concentrations of agarose (especially, 0.3% and 0.2%) were detectable after 30 days of in vivo implantation. However, the implanted biomaterials were not detectable at the macroscopical level when the FA biomaterials were grafted at the lowest concentrations of agarose, as it was the case of the F-0 and CTR groups. None of the tissues showed macroscopical signs of local complications of the implant, such as inflammation, hemorrhage, or tumorigenesis.

**Figure 5:**
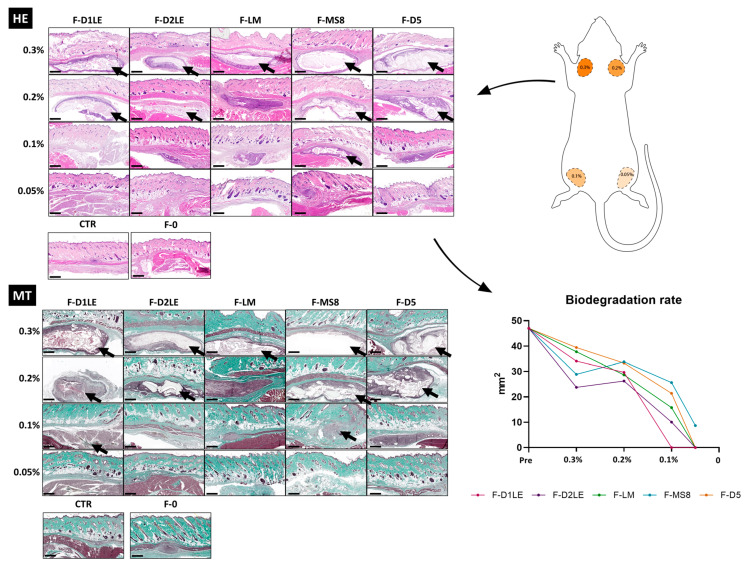
Histological analysis and biodegradation rate of the hydrogels at the implant sites in laboratory animals after 30 days of follow-up. Different types of FA biomaterials with different concentrations of agarose (0.05%, 0.1%, 0.2% and 0.3%) and fibrin biomaterials without agarose (F-0) were histologically evaluated using hematoxylin-eosin staining (HE) and Masson’s trichrome staining (MT). Animals with no grafted material were included as CTR group. F-D1LE: fibrin-agarose biomaterials containing D1LE agarose; F-D2LE: fibrin-agarose biomaterials containing D2LE agarose; F-LM: fibrin-agarose biomaterials containing LM agarose; F-D5: fibrin-agarose biomaterials containing D5 agarose. Microscopical remains of the subcutaneous implants are labeled with arrows. Scale bars: 1000 µm.

Similarly, histological evaluation of the implant site in each group of animals using hematoxylin-eosin and Masson’s trichrome staining confirmed that the FA tissues with the highest concentrations of agarose tended to remain at the implant site, whereas F-0 and FA containing the lowest agarose concentration showed no identifiable remains of the grafted biomaterial after the follow-up time (Figure 5). These results were quantified, and the biodegradation rate of the hydrogels was evaluated after 30 days of implantation (Figure 5). Our findings showed that a higher agarose concentration (0.3%) was associated with better maintenance of the hydrogel within the animal model. However, different types of agaroses had varying biodegradation rate profiles. For instance, the F-D1L2 agarose hydrogel was biodegraded completely, with no remnants observed at the concentration of 0.1%. On the other hand, the F-MS8 hydrogel was the most stable, with big remnants observed even at concentration of 0.1%. Interestingly, FA tissues containing LM agarose showed no remnants of agarose at the concentrations of 0.2%, 0.1% and 0.05%, and only some regenerative cells were found at the implant site in these study groups. For the rest of FA biomaterials, agarose was detectable at the concentration of 0.2% and, for D1LE and MS8, also for 0.1%. No signs of histological alterations, such as necrosis, infection, inflammation, or malignant transformation were detected in any of the cases, and the area of the implant was compatible with a normal tissue comparable to CTR, except for the remnants of the grafted biomaterial. In addition, we found that the remnants of the implanted material were surrounded by a thin layer of connective tissue, compatible with the presence of a narrow pseudo-capsule surrounding the implanted biomaterials (Figure 5).

### 2.5. Histochemical and Immunohistochemical Analysis of Biomaterials Grafted In Vivo in Laboratory Rats

**Figure 6:**
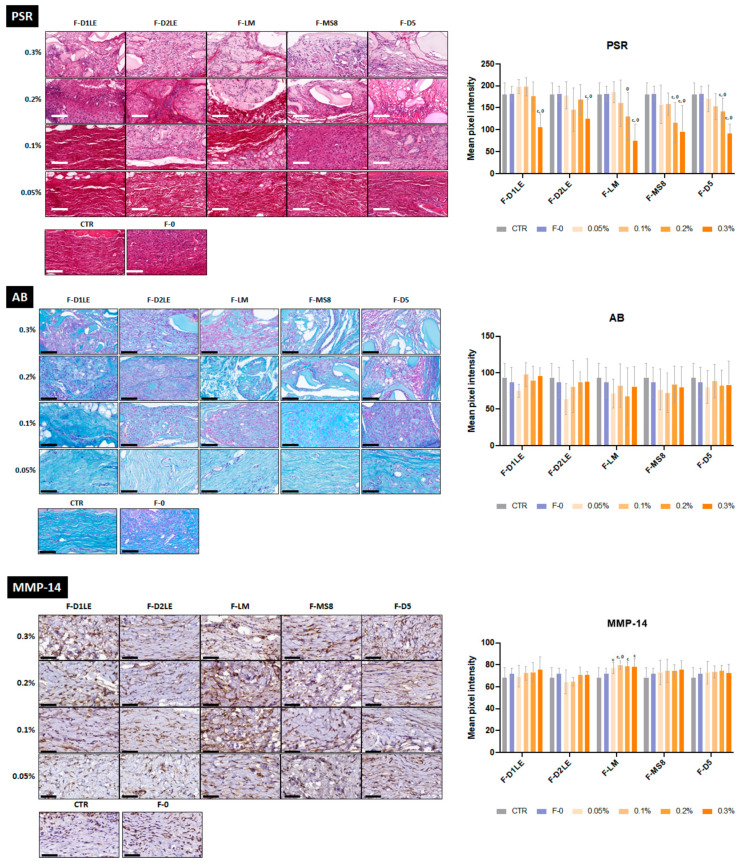
Histological and immunohistochemical analysis of the extracellular matrix remodeling of the implant sites in laboratory animals after 30 days of follow-up. Different types of FA biomaterials with different concentrations of agarose (0.05%, 0.1%, 0.2% and 0.3%) and fibrin biomaterials without agarose (F-0) were evaluated histologically using picrosirius red (PSR) and alcian blue histochemistry (AB) and immunohistochemically using anti-matrix metallopeptidase 14 (MMP-14) immunodetection. Animals with no grafted material were included as CTR group Histograms correspond to average and standard deviation results of the staining signal quantification and are shown in mean pixel intensity units. F-D1LE: fibrin-agarose biomaterials containing D1LE agarose; F-D2LE: fibrin-agarose biomaterials containing D2LE agarose; F-LM: fibrin-agarose biomaterials containing LM agarose; F-D5: fibrin-agarose biomaterials containing D5 agarose. C: differences with CTR are statistically significant; 0: differences with F-0 are statistically significant. Scale bars: 100 µm.

Analysis of the fibrillar and non-fibrillar components of tissue ECM (Figure 6 and Supplementary Table S3) showed that the grafted tissue contained variable amounts of collagen fibers and proteoglycans. Picrosirius red staining revealed that the highest concentrations of agarose tend to show lower collagen contents than CTR tissues and F-0. Statistically significant differences with the CTR group were found for all FA biomaterials containing 0.3% of agarose and, for specific agarose types (F-MS8 and F-D5), also for 0.2% *p* = <0.05 (Figure 6 and Supplementary Table S3). For the lowest concentrations of agarose, differences with CTR and F-0 were not statistically significant *p* > 0.05 (Supplementary Table S3). Quantification of tissue proteoglycans using alcian blue (Figure 6) showed very few differences among agarose types and concentrations, with non-significant differences with CTR and F-0 (Supplementary Table S3). In addition, our analysis of ECM remodeling as determined by MMP14 immunohistochemistry showed that all analyzed tissues expressed this metalloprotease. However, FA biomaterials containing LM agarose had significantly higher expression of MMP14 as compared to CTR and in all agarose concentrations, and to F-0 samples for 0.1% concentrations of agarose only (Supplementary Table S3).

**Figure 7:**
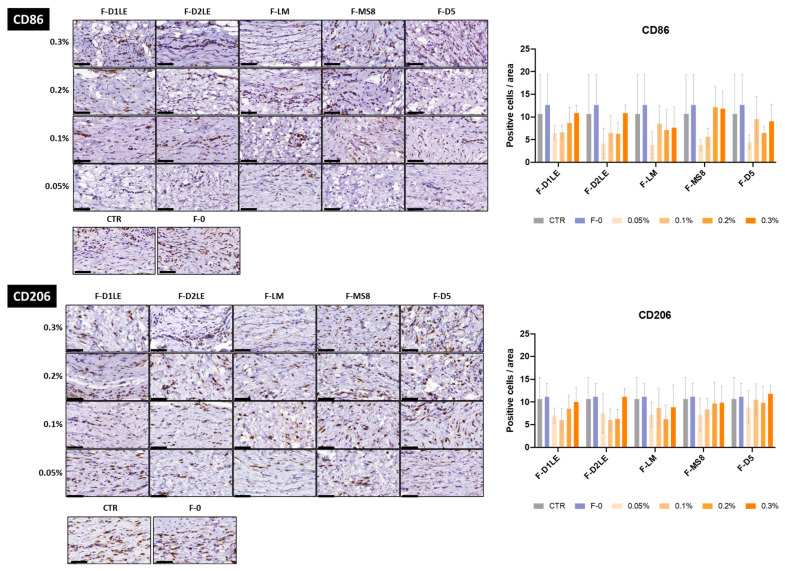
Immunohistochemical analysis of CD86 and CD206 immune cells at the implant sites of laboratory animals after 30 days of follow-up. Different types of FA biomaterials with different concentrations of agarose (0.05%, 0.1%, 0.2% and 0.3%) and fibrin biomaterials without agarose (F-0) were immunohistochemically evaluated using anti-CD86 antibodies to identify M1 pro-inflammatory macrophages and anti-CD206 antibodies to identify M2 pro-regenerative macrophages. Animals with no grafted material were included as CTR group. Histograms correspond to average and standard deviation results of the cell number quantification and are shown in number of cells per unit of area. F-D1LE: fibrin-agarose biomaterials containing D1LE agarose; F-D2LE: fibrin-agarose biomaterials containing D2LE agarose; F-LM: fibrin-agarose biomaterials containing LM agarose; F-D5: fibrin-agarose biomaterials containing D5 agarose. Scale bars: 50 µm.

Results of the immunohistochemical analysis of CD86 and CD206 expression showed that all tissues contained both the CD86-positive M1-type pro-inflammatory macrophages and the CD206-positive M2-type pro-regenerative macrophages (Figure 7). The specific analysis of each type of macrophages revealed that all samples contained a similar amount of M1-type and M2-type macrophages, with differences with CTR and F-0 being non-significant in all cases *p* > 0.05 (Figure 7).

## 3. Discussion

Development of an ideal biomaterial for clinical use is one of the main objectives of current research in tissue engineering. Although FA biomaterial showed promising results in tissue engineering of the cornea, skin, nerve, tendon, sclera and oral mucosa, the biocompatibility and biomechanical properties of this biomaterial still need to be improved [27,28,29,30,31,32]. In this regard, we recently demonstrated that different types of marine agaroses could have specific biological and biomechanical properties, suggesting that not all currently available marine agaroses polysaccharides show the same behavior both in vitro and in vivo [23]. In the present work, we evaluated several agaroses at increasing concentrations to determine their putative usefulness for the generation of human bioartificial tissues by tissue engineering. 

### 3.1. All Combinations of Fibrin and Marine-Derived Agaroses Hydrogels Are Highly Biocompatible Ex Vivo

In the first place, we found that all types of agaroses were biocompatible ex vivo, and cells cultured within the different FA biomaterials were highly viable. These results are in agreement with our previous results showing that FA biomaterials display high biocompatibility, and with our previous study showing that the five agarose types evaluated in the present work were not cytotoxic [23,38]. Although we previously found a direct correlation between the cell viability and the agarose concentration in pure agarose hydrogels, FA hydrogels seem to be very biocompatible at all concentrations evaluated here [23]. Most likely, the combination of agarose with fibrin is able to increase the biocompatibility of the FA biomaterial and reduce the slight effects of high concentrations of agarose on cell viability. These results are in line with the excellent biocompatibility of marine products such as alginate, marine-derived collagen or chitosan and hyaluronic acid that have been extensively studied for their biocompatibility and ability to support cell growth and differentiation [39]. 

### 3.2. The Agarose Type and Concentration Influence the Stiffness and Elasticity of the Resulting Hydrogels

Next, we analyzed the biomechanical properties of each type of bioartificial tissue generated in the present work to determine their potential utility for specific applications requiring definite rheological properties. Results showed that all agaroses were able to improve the biomechanical properties of pure fibrin hydrogels, as previously demonstrated [33,34]. However, we found that both the concentration and the type of agarose used in FA biomaterials influenced the biomechanical properties of these combined hydrogels. These findings are in line with those of other authors who also established that agarose concentrations of hydrogels are critical parameters in controlling their mechanical behavior [40]. Regarding the agarose type, we found that FA containing D1LE agarose showed a significant improvement of the Young’s modulus as compared to fibrin alone and to FA containing other types of agaroses. Although this modulus is a complex parameter, the stiffness of a specific biomaterial can be measured by the Young’s modulus, and the highest values of this modulus are typically associated with the highest levels of material stiffness [41]. In consequence, our results suggest that FA biomaterials containing D1LE agarose could have higher stiffness than the other types of biomaterials analyzed here. However, we found that the agarose type that was associated with a significant improvement of the biomechanical parameters that are more specifically related to tissue elasticity, such as the stress at fracture and strain at fracture, was basically the LM agarose [42]. As these parameters mainly represent the deformation capability of the biomaterial at the moment of fracture, our results may imply that FA biomaterials containing LM agarose could be more elastic than other types of FA hydrogels and that biomaterials containing fibrin alone [42]. Concerning the agarose concentration, we also found a significant association between this parameter and the different biomechanical properties. In general, we found that the material stiffness, as determined by the Young’s modulus, tended to increase with the agarose concentration, except for the FA gels containing LM agarose, whereas the elastic parameters became unchanged or tended to decrease with the agarose concentration. Interestingly, our previous analysis of pure agarose hydrogels also found a significant positive correlation of the Young’s modulus with the agarose concentration, whereas the strain at fracture tended to decrease with the concentration [23]. The tunable capability of this polysaccharide was also described for other marine-derived products such as collagen, which showed a high degree of customization using different types of crosslinking agents [43].

Although further research is needed, the results of our biomechanical analysis may have several implications. The fact that specific agarose types and concentrations resulted in FA biomaterials with definite biomechanical properties opens the door to a clinical application of each type of FA bioartificial tissue requiring specific rheological properties. In this regard, we could conclude that the biofabrication of human tissues requiring high in vivo stiffness, such as the human cornea, should use FA biomaterials containing D1LE agarose, especially at the highest concentrations. Then, tissues that are more typically elastic, such as the human skin, nerves and oral mucosa, could be preferentially reproduced using FA biomaterials with LM agarose, especially at the lowest concentrations.

### 3.3. All Types of FA Biomaterials Were Safe When Implanted In Vivo and Showed Different Biointegration Rates at the Implant Site

Biomaterials intended for future clinical use must be evaluated in laboratory animals to determine their in vivo effects in terms of biosafety and functionality [44]. Therefore, we carried out an in vivo study in laboratory rats to evaluate the systemic and local effects of each FA biomaterial. Although both short-term and long-term evaluations are important to properly evaluate the in vivo biocompatibility of an artificial graft, our previous studies about FA-based hydrogels already showed that these biomaterials are highly biocompatible and free from detectable side effects when grafted in vivo for a short period of time [35]. In the present in vivo study, we carried out a long-term time evaluation to investigate the biointegration and structural integrity over time. Future studies should be performed to evaluate the behavior of these biomaterials at shorter follow-up times. Concerning biosafety, we found that none of the hematological parameters evaluated in animals grafted with the different biomaterials was altered after 30 days of follow-up, and the use of FA and fibrin alone was comparable to control animals. Similarly, the histological analysis of five vital organs revealed no alterations in any of the study groups, with no differences with controls. In consequence, we could state that the use of the different FA biomaterials generated in the present work was safe for the animal and was not associated with a detectable adverse systemic effect in any of the cases. These results are in agreement with our previous reports showing that the in vivo implantation of hydrogels consisting of fibrin alone or the five agarose types evaluated here, is free from any detectable systemic side effects, and support the future clinical use of FA hydrogels containing the five different types of agaroses [23,35]. 

Additionally, we evaluated the local effects of each type of biomaterial to determine their potential usefulness in tissue engineering. A macroscopic analysis of the implant site revealed no signs of inflammation, necrosis, infection, rejection, or tumorigenesis, which supports the idea that the different FA biomaterials are safe for the host tissues. Moreover, our microscopic analysis confirmed the absence of any signs of complications derived from the implant and showed an ongoing process of biointegration of the bioartificial tissues at the implant sites. As expected, grafting the FA bioartificial tissues with the highest concentrations of agarose resulted in a delayed process of biointegration and biodegradation, as important parts of the graft were still detectable after the follow-up period. This phenomenon was already demonstrated for the hydrogels consisting of agarose biomaterials alone, and confirms that the most dense hydrogels are more stable and remain in place for longer periods of time [23]. The fact that certain types of agaroses were more rapidly reabsorbed even at high concentrations, especially in the case of FA containing LM, also reveals that different types of FA could behave differently once grafted in vivo [23]. Although molecular analyses should be performed to confirm or not this statement, we could hypothesize that the biomechanical properties of FA containing LM could be related to the in vivo behavior of these biomaterials. The fact that these scaffolds showed the highest elastic behavior may imply that FA with LM could be more biologically accessible to host tissue cells than other FA hydrogels that are less elastic and stiffer and, therefore, could be reabsorbed and remodeled more easily than the other types of FA. 

Interestingly, our previous studies with agarose alone revealed that the grafted material became surrounded by a thin layer of connective tissue [23]. Similarly, we also found this tissue surrounding the remnants of FA biomaterial only in those cases that were not completely reabsorbed at the time of the in vivo analysis. In both cases, this connective tissue was very thin and did not correspond to a strong pro-inflammatory fibrotic reaction. This reaction was also found by other authors testing marine-derived products. For instance, collagen-alginate hydrogels also integrate with the surrounding tissues and encapsulate in a thin layer of connective tissue after 14 weeks of follow up, which is a normal and expected response stage of the healing process [45]. 

As it is well known that pro-inflammatory responses triggered by biomaterials implanted in vivo are normally associated with a hyperfibrotic host reaction and the presence of abundant M1-type pro-inflammatory macrophages, we carried out a histochemical and immunohistochemical analysis of each type of sample [46,47,48]. First, our results revealed that the amount of collagen fibers synthetized at the grafting site was comparable or even lower than control and F-0 animals, and no differences were detected for the amount of proteoglycans at the implant site. In general, these results imply the absence of a strong fibrotic reaction driven by the FA bioartificial tissues and, therefore, a favorable healing response [49]. In addition, we found that the host tissues contained abundant M2-type pro-regenerative macrophages, although a population of M1-type pro-inflammatory macrophages was still present. Previous reports demonstrated that the initial inflammatory reaction found in tissues subjected to surgical procedures is dominated by M1-type macrophages, but these cells are progressively replaced by M2-type macrophages able to promote tissue regeneration and repair, and an alteration in this process would result in tissue damage and lack of regeneration [50,51]. In our case, the fact that the amount of both types of macrophages was similar in all study groups and did not exceed the values found in control and F-0 animals would again support the idea that the different FA biomaterials analyzed here were highly biocompatible. All these findings are in line with the high biocompatibility and biodegradability activity of marine polysaccharides described in previous studies, and reinforce the idea of their use as future advanced therapy medicinal products [52] 

Finally, our analysis of tissue remodeling as determined by MMP14 expression revealed very few differences with control and F-0 animals, except for the FA materials containing LM agarose. MMP14 is a hydrolytic metalloprotease playing an important role in ECM turnover, including degradation and regeneration, although its major function is to activate ECM degradation [53]. The similarities found between controls and tissues grafted with the different FA-based bioartificial tissues would be in agreement with the above-described results suggesting that these biomaterials are not able to significantly alter the normal physiology and turnover of grafted tissues. However, the fact that FA containing LM showed an increment of MMP14 expression could be related to the more rapid reabsorption of FA containing LM as compared to the rest of agaroses, as was also demonstrated for our previous in vivo evaluation of agarose-only biomaterials showing that LM agarose tends to became reabsorbed earlier than the other types of agaroses [23]. Most likely, the fact that LM agarose shows very low jelling temperature is associated with this phenomenon. 

The results obtained in this study demonstrated that the FA hydrogels have promising potential for tissue and organ replacement, due to their high biocompatibility and biointegration potential. Moreover, the possibility of loading the hydrogels with different biotherapeutic agents could greatly expand their potential applications, as previously demonstrated with the successful use of nanoparticles loaded with antibiotics [54]. These findings open new opportunities for further research and development in the field of tissue engineering and regenerative medicine.

## 4. Materials and Methods

In the present work, we generated FA bioartificial tissue substitutes using five different types of agaroses (D1LE, D2LE, LM, MS8 and D5) and four concentrations (0.05, 0.1, 0.2 and 0.3%) in order to determine their biocompatibility, biomechanical properties and in vivo behavior for use in tissue engineering. D1LE, D2LE, and D5 are native agaroses extracted from various species of *Gracilaria* and *Gelidium* red algae, whereas LM and MS8 are derivatized materials subjected to additional chemical modifications resulting in different physical and chemical properties. Briefly, D1LE is a typical agarose with a hysteresis of 36.6–88.3 °C and a gel strength at 1.5% of 2990 g/cm^2^; D2LE tends to gel faster than the other agarose types, and it has a hysteresis of 40.6–87.8 °C and a gel strength at 1.5% of 2310 g/cm^2^; LM has a very low gelling temperature as a result of derivatization and has a hysteresis of 26.1–64.9 °C and a gel strength at 1% of 1100 g/cm^2^; MS8 was also subjected to a derivatization process that resulted in smaller pore size and has a hysteresis of 34–78.5 °C and a gel strength at 1.5% of 3590 g/cm^2^. D5 has a hysteresis of 36.2–88.1 °C and a gel strength of 4120 g/cm^2^ at a concentration of 1.5%, enabling it to produce hydrogels with increased rigidity.

### 4.1. Cell Cultures

In the first place, we generated primary cell cultures of dermal fibroblasts as previously reported [23]. Briefly, biopsies were washed in phosphate-buffered saline -PBS- (Merck Life Science, St. Louis, MO, USA) and digested for 6 h in a 2 mg/mL solution of *Clostridium histolyticum* type I collagenase (Gibco—Thermo Fisher Scientific, Waltham, MA, USA) in Dulbecco’s Modified Eagle Medium (DMEM, Merck, Darmstadt, Germany) at 37 °C. Then, detached cells were harvested by centrifuging the digestion solution for 10 min at 1000 rpm. These cells were cultured in DMEM medium supplemented with 10% fetal bovine serum (FBS) and 1% antibiotics-antimycotics (100,000 units penicillin, 100 mg streptomycin and 250 μg amphotericin B per liter of medium; ref. A5955, Merck, Darmstadt, Germany).

### 4.2. Generation of Bioartificial Tissues Using Fibrin and Fibrin-Agarose Biomaterials

In the present work, we generated several types of bioartificial tissues containing human fibroblasts embedded within fibrin and fibrin-agarose biomaterials, as previously described [23,28]. For the tissues containing fibrin biomaterials without agarose (F-0 group), we mixed 760 µL of human plasma, 75 µL of DMEM containing 5000 detached fibroblasts, 15 µL of tranexamic acid (Amchafibrin 5 mg/mL, MEDA Pharma SL, Madrid, Spain), 50 µL of a 2% CaCl_2_ (Merck) and 100 µL of PBS, per mL of final volume. For the bioartificial tissues containing fibrin and agarose (FA biomaterials), the same mixture was used, but the 100 µL of PBS added at the final step was replaced by a melted solution of agarose in PBS. Five types of agaroses were used (D1LE, D2LE, LM, MS8 and D5, Hispanagar, Burgos, Spain) and combined with fibrin (F-D1LE, F-D2LE, F-LM, F-MS8 and F-D5 FA hydrogels, respectively) at different final concentrations (0.05%, 0.1%, 0.2% and 0.3% (*w*/*v*)) following previous published studies that showed variation in hydrogel biomechanical and rheological properties [33,34]. For the in vivo analyses, the same types of samples were generated without cells (acellular biomaterials) (see Supplementary Figure S1). In all cases, the mixture was carefully aliquoted in 6-well plates (Corning Life Sciences, Corning, NY, USA) and incubated in a 37 °C cell incubator to favor biomaterial polymerization and jellification. Then, bioartificial tissues were extracted from the culture plates and subjected to plastic compression nanostructuration as previously described [30,33,34].

### 4.3. Cell Viability Analysis

To determine the ex vivo biocompatibility of the different biomaterials evaluated here, we evaluated the viability of the cells immersed within each biomaterial using LIVE/DEAD assay kits (Life Technologies, Carlsbad, CA, USA), as previously described [23]. In brief, a fragment of approximately 3 mm^3^ was obtained from each bioartificial tissue generated 48 h before and incubated for 5 min in a mixture of calcein and propidium iodide following the manufacturer’s instructions. Tissues were then washed in PBS, deposited on a glass slide, coverslipped and observed using a Nikon Eclipse i90 microscope (Nikon, Tokyo, Japan). Fluorescence images were used in the green and red channels to identify live and dead cells, respectively. The percentage of live cells was calculated in each sample. Cells cultured on culture plates without biomaterial were analyzed as positive controls (CTR+), and cells cultured on culture plates and treated with 70% ethanol for 1 min were used as negative controls (CTR-). All viability experiments were carried out using sextuplicates (*n* = 6).

### 4.4. Biomechanical Evaluation

Assessment of the biomechanical properties of the different bioartificial tissues generated in the present work was carried out using a Model 5943 biomechanical analyzer (Instron Corp, Norwood, MA, USA) with BlueHill 3 Material Testing software. Each sample with a length of 3 cm and a width of 1cm was fixed between both plates of the analyzer, leaving 1 cm between the plates. Samples were then subjected to a controlled tension stress with a constant strain rate of 5 mm/min until the material was broken. Then, the Young’s modulus was calculated as the tangent modulus of the linear portion of the stress-strain curve. The stress at fracture and the strain at fracture were calculated by selecting the point of the stress-strain curve where the material broke, and the break load was the force at which this fracture occurred. All samples were measured using exactly the same conditions, and 6 samples of each type were analyzed (*n* = 6).

### 4.5. In Vivo Analysis

To evaluate the in vivo behavior of each type of material, acellular biomaterials (F-0, F-D1LE, F-D2LE, F-LM, F-MS8 and F-D5) were subcutaneously implanted in 12-week-old male Wistar rats (*n* = 4 per condition) after approval by the Animal Experimentation Ethics Committee (*Comité de Ética y Experimentación Animal, CEEA*), protocol code 19/04/2021/053. First, animals were deeply anesthetized using ketamine and acepromazine (Boehringer Ingelheim, Ingelheim am Rhein, Germany). Then, a subcutaneous pouch was surgically dissected at four different places at the back of the Wistar rats, near the origin of the four limbs, with a distance of at least 4 cm among pouches to minimize the impact of any movement or pressure on the implants, and to provide a consistent and controlled environment for the implantation of the FA biomaterials (Figure 5 and Supplementary Figure S1). Then, a disk of 8 mm of diameter of each concentration (0.05%, 0.1%, 0.2% and 0.3%) of a specific biomaterial (F-D1LE, F-D2LE, F-LM, F-MS8 or F-D5) was implanted in each pouch. This way, each animal received the four concentrations of the same biomaterial in different subcutaneous areas. For animals assigned to the F-0 group, fibrin biomaterials devoid of agarose were grafted at the four areas of the same animal. Finally, the skin was repaired using absorbable surgical material. As controls, 4 rats were subjected to the same surgical procedure, but no materials were grafted in the generated pouches (CTR group). A total of 28 animals were included in the study, and all animals were euthanatized under general anesthesia 30 days after the surgical procedure. 

To assess the systemic effects of each type of biomaterial to determine their biocompatibility, five major organs (heart, spleen, liver, kidney and lung), along with 1 mL of peripheral blood, were harvested from each animal at the moment of the euthanasia. Blood samples were analyzed for several key hematological and biochemical parameters using a Sysmex KX-21N automatic analyzer and a clinical chemistry analyzer Cobas c311 (Roche, Basel, Switzerland).

### 4.6. Histology, Histochemistry and Immunohistochemistry

At the moment of the euthanasia, the local site of the implant was surgically dissected and photographed. Then, local implant sites and the five major organs extracted from each rat were fixed in formalin. These histological samples were dehydrated and embedded in paraffin following routine protocols. Tissue sections were obtained and stained with hematoxylin-eosin and Masson’s trichrome for morphological analysis. To identify relevant fibrillar and non-fibrillar components of the tissue ECM, tissue sections were subjected to histochemical methods using picrosirius red for collagen fibers and alcian blue for proteoglycans, as previously described [55,56]. Detection of M1-type pro-inflammatory macrophages and M2-type pro-regenerative macrophages was performed by immunohistochemistry for the CD86 and CD206 markers, respectively, whereas ECM remodeling was assessed by immunohistochemistry for the metalloproteinase MMP14. In brief, sections were dewaxed and rehydrated, and citrate buffer pH 6 was used for antigen retrieval (Merck). Then, H_2_O_2_ (Panreac Química S.L.U., Barcelona, Spain) was used to quench endogenous peroxidases and casein and horse serum (Vector laboratories, Burlingame, CA, USA) were used for prehybridization. Tissues were then incubated overnight with a 1:200 and 1:800 dilution of anti-CD86 or anti-CD206 primary antibodies, respectively (Abcam, Cambridge, UK). After washing in PBS, samples were incubated with a ready-to-use peroxidase-labeled secondary antibody solution (Vector Laboratories, Burlingame, CA, USA). Then, tissue sections were washed in PBS, incubated in a diaminobenzidine solution (Vector Laboratories, Burlingame, CA, USA) and counterstained with Harry’s hematoxylin (Thermo Fisher Scientific, Waltham, MA, USA).

### 4.7. Quantification and Statistical Analysis

The biodegradation rate of the hydrogels was calculated by quantifying the area occupied by the graft in the histological sections stained with hematoxylin and eosin. This quantification was carried out using the software ImageJ (version 1.53k, National Institutes of Health, Bethesda, MD, USA), which allowed us to determine the remaining graft area after 30 days of implantation. 

Results corresponding to the analysis of ECM composition (picrosirius red and alcian blue histochemistry and MMP14 immunohistochemistry) were quantitatively analyzed by determining the signal intensity of each staining method using the software ImageJ (National Institutes of Health, Bethesda, MD, USA), as previously described [57]. In brief, 10 dots were randomly selected on each histological image and the signal intensity was automatically calculated by the program as intensity units. For CD86 and CD206 immunohistochemistry, the number of cells showing a positive signal was determined in each area of each sample. In this case, we selected random square areas of 70 × 70 µm in each histological image, and the number of positive cells within the 4900 µm^2^ square was analyzed. 

For each variable, we calculated averages and standard deviations for each specific group. For cell viability and for the biomechanical parameters, we also calculated the averages and standard deviation of global groups of samples (for example, all samples containing a specific type of agarose, regardless of the concentration). 

Then, each variable was analyzed using the Shapiro–Wilk Test to determine the normality of each distribution. For variables fulfilling the normality and parametricity criteria (quantitative results of collagen, proteoglycans, M1 and M2-type macrophages and MMP14 activity), comparisons were carried out using ANOVA with the Tukey post-hoc test. For distributions not fulfilling these criteria (cell viability, biomechanical properties, and hematological parameters), the non-parametric Mann–Whitney test was used. Statistical comparisons were performed using Real Statistics (Dr. Charles Zaiontz, Purdue University, West Lafayette, IN, USA). 

## 5. Conclusions

In conclusion, this study demonstrated that marine agarose polysaccharides can improve the biomechanical properties of fibrin hydrogels, resulting in highly biocompatible FA biomaterials both ex vivo and in vivo. The possibility of customizing the biomechanical properties of these materials by adjusting the type and concentration of agarose used, according to the specific needs of the recipient tissue, makes them promising candidates for the clinical regeneration of different human tissues, such as the skin, cornea, oral mucosa, palate and nerve. Moreover, the preclinical characterization and quality control analysis carried out in this study provide a crucial requirement for future clinical translation. Overall, the findings suggest that FA biomaterials can drive a proregenerative process devoid of a strong inflammatory response. This supports their potential use in tissue regeneration applications with the possibility of developing FA biomaterials with specific behavior derived from the type and concentration of agarose used in the FA mixture. 

## Data Availability

The data presented in this study are available on request from the corresponding author.

## Supplementary Materials

The following supporting information can be downloaded at: https://www.mdpi.com/article/10.3390/md21030187/s1, Figure S1: Schematic drawing of the manufacturing and grafting process of fibrin-agarose hydrogels. F-0: fibrin hydrogels de-void of agarose; FA: all hydrogels containing fibrin and agarose, regardless the type and concentration of agarose. F-D1LE: fi-brin-agarose biomaterials containing D1LE agarose; F-D2LE: fibrin-agarose biomaterials containing D21LE agarose; F-LM: fi-brin-agarose biomaterials containing LM agarose; F-MS8: fibrin-agarose biomaterials containing MS8 agarose; F-D5: fibrin-agarose biomaterials containing D5 agarose. Scale bar: 4 mm; Table S1: Analysis of cell viability and biomechanical properties of each type of sample. Values are shown as means ± standard deviations of each analysis group. F-0: fibrin hydrogels devoid of agarose; FA: all hydrogels containing fibrin and agarose, re-gardless the type and concentration of agarose. F-D1LE: fibrin-agarose biomaterials containing D1LE agarose; F-D2LE: fibrin-agarose biomaterials containing D21LE agarose; F-LM: fibrin-agarose biomaterials containing LM agarose; F-MS8: fibrin-agarose biomaterials containing MS8 agarose; F-D5: fibrin-agarose biomaterials containing D5 agarose; Table S2: Statistical p values for the comparison of the cell viability and biomechanical properties among different groups of samples (global and specific groups). For the comparisons of global groups, analyses were carried out among types of bio-materials (regardless the concentration of agarose) and among concentrations (regardless the type of agarose). Statistically sig-nificant results are highlighted with asterisks (*). F-0: fibrin hydrogels devoid of agarose; FA: all hydrogels containing fibrin and agarose, regardless the type and concentration of agarose. F-D1LE: fibrin-agarose biomaterials containing D1LE agarose; F-D2LE: fibrin-agarose biomaterials containing D21LE agarose; F-LM: fibrin-agarose biomaterials containing LM agarose; F-MS8: fibrin-agarose biomaterials containing MS8 agarose; F-D5: fibrin-agarose biomaterials containing D5 agarose; Table S3: Statistical p values for the comparison of the histochemical and immunohistochemical staining among different sam-ples versus control group and fibrin hydrogels. Statistically significant results are highlighted with asterisks (*). F-0: fibrin hy-drogels devoid of agarose; FA: all hydrogels containing fibrin and agarose, regardless the type and concentration of agarose. F-D1LE: fibrin-agarose biomaterials containing D1LE agarose; F-D2LE: fibrin-agarose biomaterials containing D21LE agarose; F-LM: fibrin-agarose biomaterials containing LM agarose; F-MS8: fibrin-agarose biomaterials containing MS8 agarose; F-D5: fi-brin-agarose biomaterials containing D5 agarose.
