# Supplementary material for: Fibrin and Marine-Derived Agaroses for the Generation of Human Bioartificial Tissues: An Ex Vivo and In Vivo Study
Source: Mar Drugs. 2023 Mar 17;21(3):187. doi: 10.3390/md21030187 (PMC10058299; doi:10.3390/md21030187)
Supplement: Supplementary file 1 [file marinedrugs-21-00187-s001.zip › Supplementary Tables S1--S3.pdf]

Supplementary Table S1. Analysis of cell viability and biomechanical properties of each type of sample. Values are shown as means ± standard deviations of each analysis group. F-0: fibrin hydrogels devoid of agarose; FA: all hydrogels containing fibrin and agarose, regardless the type and concentration of agarose. F-D1LE: fibrin-agarose biomaterials containing D1LE agarose; F-D2LE: fibrin-agarose biomaterials containing D21LE agarose; F-LM: fibrin-agarose biomaterials containing LM agarose; F-MS8: fibrin-agarose biomaterials containing MS8 agarose; F-D5: fibrin-agarose biomaterials containing D5 agarose.

Sample Type	Agarose Concentration	Cell Viability (%)	Young Modulus (MPa)	Stress At Fracture (MPa)	Strain At Fracture (%)	Break Load (N)
F-0	0%	100±3.14	0.1701±0.0117	0.4927±0.1775	221.9467±23.1921	0.2463±0.0888
FA	All concentations	98.24±4.35	0.2987±0.1249	0.5012±0.2370	261.1046±76.4858	0.3038±0.1420
F-D1LE	All concentations	99.13±2.36	0.3788±0.1681	0.4327±0.1745	210.7695±43.6563	0.2596±0.1047
F-D2LE	All concentations	99.27±2.48	0.2763±0.0640	0.4741±0.1253	251.1447±41.4778	0.3036±0.0753
F-LM	All concentations	97.87±3.41	0.2885±0.1188	0.6562±0.2764	325.8786±73.3308	0.3937±0.1658
F-MS8	All concentations	97.53±6.71	0.2589±0.0878	0.4311±0.2709	236.2120±71.9679	0.2587±0.1625
F-D5	All concentations	97.4±5.11	0.2852±0.1206	0.5181±0.2280	284.4259±83.7706	0.3109±0.1368
All types of agarose	0.05%	95.95±6.92	0.2862±0.1178	0.5912±0.2397	277.0291±62.5946	0.3547±0.1438
	0.10%	98.31±3.66	0.2914±0.0881	0.5986±0.2060	281.4055±72.2857	0.3592±0.1236
	0.20%	99.08±2.48	0.2757±0.0811	0.4653±0.2086	255.2131±85.5107	0.2906±0.1260
	0.30%	99.62±1.46	0.2816±0.2236	0.2161±0.1931	161.1427±116.9692	0.1297±0.1159
F-D1LE	0.05%	97.67±3.63	0.2800±0.1262	0.4536±0.1238	238.0654±61.8265	0.2722±0.0743
	0.10%	98.85±2.83	0.3822±0.1094	0.5105±0.2344	220.4391±29.9518	0.3063±0.1407
	0.20%	100±0	0.2921±0.0588	0.3462±0.1468	189.3716±38.8563	0.2077±0.0881
	0.30%	100±0	0.5611±0.1948	0.4204±0.1767	195.2020±26.2877	0.2522±0.1060
F-D2LE	0.05%	98.37±3.98	0.2641±0.0447	0.4575±0.0617	253.8015±18.1873	0.2745±0.0370
	0.10%	98.72±3.14	0.2454±0.0267	0.5044±0.1574	254.4134±71.0948	0.3027±0.0944
	0.20%	100±0	0.3194±0.0875	0.4605±0.1522	245.2191±20.0176	0.3336±0.0829
	0.30%	100±0	-	-	-	-
F-LM	0.05%	95.7±4.42	0.4186±0.1575	0.9074±0.0858	347.9151±43.4136	0.5444±0.0515
	0.10%	98.95±2.56	0.2713±0.0737	0.6589±0.2633	293.3282±88.6031	0.3953±0.1580
	0.20%	97.69±3.73	0.2208±0.0468	0.6307±0.2707	342.5928±74.5234	0.3784±0.1624
	0.30%	99.13±2.13	0.2433±0.0618	0.3137±0.0318	316.5785±91.6292	0.1882±0.0191
F-MS8	0.05%	93±11.97	0.2551±0.0798	0.5396±0.2649	246.8549±52.6898	0.3237±0.1589
	0.10%	97.1257±4.9441	0.2551±0.0669	0.6763±0.2162	299.0610±60.4168	0.4058±0.1297
	0.20%	100±0	0.2473±0.0828	0.3192±0.1892	178.3862±55.1791	0.1915±0.1135
	0.30%	100±0	0.2782±0.1309	0.1895±0.1163	220.5459±71.9095	0.1137±0.0698
F-D5	0.05%	95.0199±7.9788	0.2132±0.046	0.5981±0.2629	298.5086±61.3628	0.3588±0.1577
	0.10%	97.9186±5.0984	0.3033±0.0881	0.6429±0.1277	339.7860±53.7112	0.3857±0.0766
	0.20%	97.6918±3.7337	0.2990±0.1004	0.5699±0.0998	320.4957±72.2485	0.3420±0.0599
	0.30%	98.9548±2.5602	0.3253±0.1953	0.2616±0.1878	178.9132±39.6732	0.1569±0.1127

Supplementary Table S2. Statistical p values for the comparison of the cell viability and biomechanical properties among different groups of samples (global and specific groups). For the comparisons of global groups, analyses were carried out among types of biomaterials (regardless the concentration of agarose) and among concentrations (regardless the type of agarose). Statistically significant results are highlighted with asterisks (*). F-0: fibrin hydrogels devoid of agarose; FA: all hydrogels containing fibrin and agarose, regardless the type and concentration of agarose. F-D1LE: fibrin-agarose biomaterials containing D1LE agarose; F-D2LE: fibrin-agarose biomaterials containing D21LE agarose; F-LM: fibrin-agarose biomaterials containing LM agarose; F-MS8: fibrin-agarose biomaterials containing MS8 agarose; F-D5: fibrin-agarose biomaterials containing D5 agarose

		CELL VIABILITY (%)	YOUNG MODULUS (Mpa)	STRESS AT FRACTURE (MPa)	STRAIN AT FRACTURE (%)	BREAK LOAD (N)
**All agarose concentrations within each type of fibrin-agarose biomaterial (global groups)**	**F-0 vs. F-D1LE**	0.12860	0.00022*	0.40213	0.46221	0.97985
	**F-0 vs. F-D2LE**	0.15777	0.00001*	0.67292	0.05586	0.27959
	**F-0 vs. F-LM**	0.07311	0.00057*	0.09955	0.00158*	0.05166
	**F-0 vs. F-MS8**	0.11558	0.00753*	0.56053	0.82024	0.97985
	**F-0 vs. F-D5**	0.07311	0.00051*	0.74262	0.07312	0.25063
	**F-D1LE vs. F-D2LE**	0.84613	0.04809*	0.31904	0.00265*	0.13152
	**F-D1LE vs. F-LM**	0.26377	0.03742*	0.00539*	<0.00001*	0.00539*
	**F-D1LE vs. F-MS8**	0.76704	0.00676*	0.67527	0.26377	0.67527
	**F-D1LE vs. F-D5**	0.41884	0.04149*	0.13051	0.00149*	0.13051
	**F-D2LE vs. F-LM**	0.17185	0.79180	0.04482*	0.00055*	0.10041
	**F-D2LE vs. F-MS8**	0.60217	0.28339	0.28339	0.41286	0.13824
	**F-D2LE vs. F-D5**	0.32038	0.69669	0.34434	0.11282	0.62375
	**F-LM vs. F-MS8**	0.44288	0.56008	0.00825*	0.00021*	0.00825*
	**F-LM vs. F-D5**	0.84613	0.79845	0.08433	0.09271	0.08433
	**F-MS8 vs. F-D5**	0.67526	0.66038	0.17186	0.04828*	0.17186
**All agarose types within each concentration of fibrin-agarose biomaterial (global groups)**	**0.05% vs. F-0**	0.00022*	<0.00001*	0.24180	0.00002*	0.00918*
	**0.1% vs. F-0**	0.00234*	<0.00001*	0.01759*	0.00069*	0.00007*
	**0.2% vs. F-0**	0.0073*	<0.00001*	0.46707	0.07718	0.18718
	**0.3% vs. F-0**	0.01551*	0.07718	<0.00001*	0.00161*	0.00004*
	**0.05% vs. 0.1%**	0.24781	0.59200	0.65434	0.87755	0.65434
	**0.05% vs. 0.2%**	0.09349	0.91237	0.11585	0.15469	0.21876
	**0.05% vs. 0.3%**	0.03712*	0.66498	<0.00001*	0.00001*	<0.00001*
	**0.1% vs. 0.2%**	0.63326	0.54220	0.0073*	0.18718	0.01990*
	**0.1% vs. 0.3%**	0.35042	0.58188	<0.00001*	0.00002*	<0.00001*
	**0.2% vs. 0.3%**	0.65434	0.71915	0.00001*	0.00161*	<0.00001*
**F-D1LE**	**0.05% vs. F-0**	0.13203	0.06494	0.69913	0.81818	0.81818
	**0.1% vs. F-0**	0.24025	0.00866*	0.93723	0.81818	0.39394
	**0.2% vs. F-0**	0.39393	0.00216*	0.24026	0.17965	0.39394
	**0.3% vs. F-0**	0.39393	0.00216*	0.39394	0.13203	0.93723
	**0.05% vs. 0.1%**	0.69913	0.24026	0.81818	0.58874	0.81818
	**0.05% vs. 0.2%**	0.39393	0.48485	0.30952	0.09307	0.30952
	**0.05% vs. 0.3%**	0.39393	0.02597*	0.69913	0.24026	0.69913
	**0.1% vs. 0.2%**	0.69913	0.06494	0.17965	0.17965	0.17965
	**0.1% vs. 0.3%**	0.69913	0.09307	0.58874	0.17965	0.58874
	**0.2% vs. 0.3%**	0.93722	0.02597*	0.48485	0.93723	0.48485
**F-D2LE**	**0.05% vs. F-0**	0.24025	0.00216*	0.58874	0.00866*	0.69913
	**0.1% vs. F-0**	0.24025	0.00216*	0.81818	0.81818	0.39394
	**0.2% vs. F-0**	0.39393	0.00216*	0.48485	0.09307	0.24026
	**0.3% vs. F-0**	0.39393	-	-	-	-
	**0.05% vs. 0.1%**	0.93722	0.58874	0.93723	0.93723	0.93723
	**0.05% vs. 0.2%**	0.69913	0.24026	0.69913	0.93723	0.17965
	**0.05% vs. 0.3%**	0.69913	-	-	-	-
	**0.1% vs. 0.2%**	0.69913	0.24026	0.93723	0.93723	0.48485
	**0.1% vs. 0.3%**	0.69913	-	-	-	-
	**0.2% vs. 0.3%**	0.93722	-	-	-	-
**F-LM**	**0.05% vs. F-0**	0.09307	0.00216*	0.00216*	0.00216*	0.00216*
	**0.1% vs. F-0**	0.24025	0.00433*	0.13203	0.13203	0.06494
	**0.2% vs. F-0**	0.17965	0.02597*	0.30952	0.00216*	0.17965

	0.3% vs. F-0	0.30952	0.06494	0.09307	0.93723	0.13203
	0.05% vs. 0.1%	0.24025	0.09307	0.01515*	0.24026	0.01515*
	0.05% vs. 0.2%	0.39393	0.02597*	0.06494	0.93723	0.06494
	0.05% vs. 0.3%	0.13203	0.13203	0.00216*	0.24026	0.00216*
	0.1% vs. 0.2%	0.69913	0.17965	0.58874	0.30952	0.58874
	0.1% vs. 0.3%	0.93722	0.48485	0.02597*	0.58874	0.02597*
	0.2% vs. 0.3%	0.58874	0.39394	0.00433*	0.24026	0.00433*
**F-MS8**	**0.05% vs. F-0**	0.13203	0.02597*	0.93723	0.30952	0.58874
	**0.1% vs. F-0**	0.17965	0.00216*	0.09307	0.04113*	0.02597*
	**0.2% vs. F-0**	0.39393	0.06494	0.17965	0.09307	0.48485
	**0.3% vs. F-0**	0.39393	0.39394	0.02597*	0.58874	0.02597*
	**0.05% vs. 0.1%**	0.81818	0.93723	0.30952	0.13203	0.30952
	**0.05% vs. 0.2%**	0.39393	0.58874	0.17965	0.06494	0.17965
	**0.05% vs. 0.3%**	0.39393	0.93723	0.00866*	0.48485	0.00866*
	**0.1% vs. 0.2%**	0.39393	0.93723	0.01515*	0.00866*	0.01515*
	**0.1% vs. 0.3%**	0.39393	0.58874	0.00866*	0.13203	0.00866*
	**0.2% vs. 0.3%**	0.93722	0.69913	0.30952	0.17965	0.30952
**F-D5**	**0.05% vs. F-0**	0.13203	0.06494	0.48485	0.04113*	0.24026
	**0.1% vs. F-0**	0.24025	0.00216*	0.17965	0.00216*	0.02597*
	**0.2% vs. F-0**	0.17965	0.00866*	0.48485	0.01515*	0.06494
	**0.3% vs. F-0**	0.24025	0.39394	0.06494	0.09307	0.09307
	**0.05% vs. 0.1%**	0.69913	0.13203	0.69913	0.39394	0.69913
	**0.05% vs. 0.2%**	0.81818	0.24026	0.81818	0.69913	0.81818
	**0.05% vs. 0.3%**	0.58874	0.93723	0.04113*	0.00866*	0.04113*
	**0.1% vs. 0.2%**	0.81818	0.93723	0.39394	0.93723	0.39394
	**0.1% vs. 0.3%**	0.93722	0.93723	0.01515*	0.00216*	0.01515*
	**0.2% vs. 0.3%**	0.69913	0.93723	0.00866*	0.00433*	0.00866*
**0.05% agarose concentration**	**F-D1LE vs. F-D2LE**	0.81818	0.93723	0.69913	0.58874	0.69913
	**F-D1LE vs. F-LM**	0.58874	0.13203	0.00216*	0.01515*	0.00216*
	**F-D1LE vs. F-MS8**	0.81818	0.93723	0.93723	0.81818	0.93723
	**F-D1LE vs. F-D5**	0.81818	0.48485	0.39394	0.17965	0.39394
	**F-D2LE vs. F-LM**	0.24025	0.06494	0.00216*	0.00433*	0.00216*
	**F-D2LE vs. F-MS8**	0.58874	0.69913	0.93723	0.69913	0.93723
	**F-D2LE vs. F-D5**	0.58874	0.09307	0.48485	0.24026	0.48485
	**F-LM vs. F-MS8**	0.81818	0.06494	0.06494	0.00866*	0.06494
	**F-LM vs. F-D5**	0.69913	0.00866*	0.09307	0.17965	0.09307
	**F-MS8 vs. F-D5**	0.93722	0.30952	0.69913	0.30952	0.69913
**0.1% agarose concentration**	**F-D1LE vs. F-D2LE**	0.93722	0.06494	0.93723	0.81818	0.93723
	**F-D1LE vs. F-LM**	0.93722	0.06494	0.30952	0.13203	0.30952
	**F-D1LE vs. F-MS8**	0.69913	0.06494	0.17965	0.02597*	0.17965
	**F-D1LE vs. F-D5**	0.93722	0.17965	0.30952	0.00216*	0.30952
	**F-D2LE vs. F-LM**	0.93722	0.69913	0.17965	0.48485	0.17965
	**F-D2LE vs. F-MS8**	0.69913	0.81818	0.17965	0.30952	0.17965
	**F-D2LE vs. F-D5**	0.93722	0.30952	0.24026	0.09307	0.24026
	**F-LM vs. F-MS8**	0.69913	0.81818	0.93723	0.93723	0.93723
	**F-LM vs. F-D5**	0.93722	0.39394	0.58874	0.39394	0.58874
	**F-MS8 vs. F-D5**	0.81818	0.39394	0.58874	0.24026	0.58874
**0.2% agarose concentration**	**F-D1LE vs. F-D2LE**	0.93722	0.69913	0.17965	0.01515*	0.04113*
	**F-D1LE vs. F-LM**	0.39393	0.04113*	0.06494	0.00216*	0.06494
	**F-D1LE vs. F-MS8**	0.93722	0.39394	0.69913	0.39394	0.69913
	**F-D1LE vs. F-D5**	0.39393	0.93723	0.01515*	0.00433*	0.01515*
	**F-D2LE vs. F-LM**	0.39393	0.04113*	0.39394	0.01515*	0.93723
	**F-D2LE vs. F-MS8**	0.93722	0.13203	0.30952	0.06494	0.09307
	**F-D2LE vs. F-D5**	0.39393	0.93723	0.39394	0.04113*	0.93723
	**F-LM vs. F-MS8**	0.39393	0.48485	0.02597*	0.00433*	0.02597*
	**F-LM vs. F-D5**	0.93722	0.30952	0.81818	0.81818	0.81818
	**F-MS8 vs. F-D5**	0.39393	0.48485	0.02597*	0.00433*	0.02597*
**0.3% agarose concentration**	**F-D1LE vs. F-D2LE**	0.93722	-	-	-	-
	**F-D1LE vs. F-LM**	0.69913	0.01515*	0.35238	0.03810*	0.35238
	**F-D1LE vs. F-MS8**	0.93722	0.01515*	0.04113*	0.69913	0.04113*
	**F-D1LE vs. F-D5**	0.69913	0.09307	0.09307	0.24026	0.09307

**F-D2LE vs. F-LM**	0.69913	-	-	-	-
**F-D2LE vs. F-MS8**	0.93722	-	-	-	-
**F-D2LE vs. F-D5**	0.69913	-	-	-	-
**F-LM vs. F-MS8**	0.69913	0.69913	0.11429	0.17143	0.11429
**F-LM vs. F-D5**	0.93722	0.93723	0.47619	0.01905*	0.47619
**F-MS8 vs. F-D5**	0.69913	0.58874	0.58874	0.24026	0.58874

Supplementary Table S3. Statistical p values for the comparison of the histochemical and immunohistochemical staining among different samples versus control group and fibrin hydrogels. Statistically significant results are highlighted with asterisks (*). F-0: fibrin hydrogels devoid of agarose; FA: all hydrogels containing fibrin and agarose, regardless the type and concentration of agarose. F-D1LE: fibrin-agarose biomaterials containing D1LE agarose; F-D2LE: fibrin-agarose biomaterials containing D21LE agarose; F-LM: fibrin-agarose biomaterials containing LM agarose; F-MS8: fibrin-agarose biomaterials containing MS8 agarose; F-D5: fibrin-agarose biomaterials containing D5 agarose

		PICROSIRIUS RED	ALCIAN BLUE	MMP14	CD86	CD206
	**0.05% vs. F-0**	0.67627	0.66782	0.99283	0.25808	0.23204
	**0.1% vs. F-0**	0.720164	0.70861	0.99999	0.28503	0.07954
	**0.2% vs. F-0**	0.99618	0.99903	0.99924	0.70264	0.69030
	**0.3% vs. F-0**	0.00000*	0.87408	0.93992	0.98495	0.98698
**D1LE**	**CTR vs. F-0**	0.99992	0.94468	0.95323	0.97789	0.99977
	**CTR vs. 0.05%**	0.53278	0.17549	0.99966	0.66635	0.36085
	**CTR vs. 0.1%**	0.57902	0.99471	0.91651	0.70264	0.13971
	**CTR vs. 0.2%**	0.99980	0.99471	0.82652	0.97789	0.83930
	**CTR vs. 0.3%**	0.00000*	0.99994	0.48455	1.00000	0.99906
	**0.05% vs. F-0**	0.999838	0.325850	0.191671	0.067429	0.389224
	**0.1% vs. F-0**	0.221595	0.996204	0.233849	0.309696	0.092955
	**0.2% vs. F-0**	0.962280	1.000000	0.999568	0.282619	0.133617
	**0.3% vs. F-0**	0.008459*	0.999999	0.999431	0.988063	1.000000
**D2LE**	**CTR vs. F-0**	0.999986	0.989836	0.891524	0.982386	0.999796
	**CTR vs. 0.05%**	0.999998	0.099257	0.792508	0.257129	0.548315
	**CTR vs. 0.1%**	0.292956	0.877611	0.845076	0.711859	0.158832
	**CTR vs. 0.2%**	0.985042	0.993355	0.973792	0.677663	0.220260
	**CTR vs. 0.3%**	0.01331*	0.995511	0.976446	1.000000	0.999796
	**0.05% vs. F-0**	0.999954	0.805083	0.370240	0.091787	0.499527
	**0.1% vs. F-0**	0.802965	0.999288	0.047878*	0.780640	0.875117
	**0.2% vs. F-0**	0.042474*	0.628928	0.145490	0.529283	0.262275
	**0.3% vs. F-0**	0.000001*	0.997397	0.198126	0.626977	0.903306
**LM**	**CTR vs. F-0**	0.999990	0.992953	0.814252	0.988174	0.999919
	**CTR vs. 0.05%**	0.999316	0.459844	0.025360*	0.295808	0.637185
	**CTR vs. 0.1%**	0.871984	0.941855	0.001289*	0.983078	0.947290
	**CTR vs. 0.2%**	0.061116	0.291890	0.005826*	0.879428	0.371047
	**CTR vs. 0.3%**	0.000001*	0.909415	0.009196*	0.933068	0.963254
**MS8**	**0.05% vs. F-0**	0.723208	0.956470	0.998967	0.058828	0.468489

	**0.1% vs. F-0**	0.753252	0.804197	0.971761	0.205186	0.788273
	**0.2% vs. F-0**	0.004884*	0.999933	0.973739	0.999979	0.982551
	**0.3% vs. F-0**	0.000108*	0.992417	0.901035	0.999741	0.989716
	**CTR vs. F-0**	0.999991	0.989984	0.948428	0.983699	0.999907
	**CTR vs. 0.05%**	0.803312	0.691591	0.804303	0.226620	0.609290
	**CTR vs. 0.1%**	0.829251	0.431392	0.567924	0.554359	0.892383
	**CTR vs. 0.2%**	0.007498*	0.961968	0.576134	0.995626	0.997301
	**CTR vs. 0.3%**	0.000177*	0.843920	0.396486	0.998674	0.998871
	**0.05% vs. F-0**	0.930422	0.990045	0.999494	0.101285	0.850421
	**0.1% vs. F-0**	0.144177	0.999964	0.996269	0.895543	0.999404
	**0.2% vs. F-0**	0.011574*	0.998778	0.943754	0.338749	0.984506
	**0.3% vs. F-0**	0.000000*	0.999633	0.999908	0.823788	0.999404
**D5**	**CTR vs. F-0**	0.999936	0.986889	0.912450	0.984433	0.999854
	**CTR vs. 0.05%**	0.975880	0.808533	0.764363	0.338749	0.939866
	**CTR vs. 0.1%**	0.219739	0.997401	0.664237	0.998738	0.999999
	**CTR vs. 0.2%**	0.021031*	0.904063	0.404573	0.734685	0.998252
	**CTR vs. 0.3%**	0.000000*	0.931546	0.815401	0.993197	0.991528
