# Supplementary figures and images for: Fibrin and Marine-Derived Agaroses for the Generation of Human Bioartificial Tissues: An Ex Vivo and In Vivo Study
Source: Mar Drugs. 2023 Mar 17;21(3):187. doi: 10.3390/md21030187 (PMC10058299; doi:10.3390/md21030187)

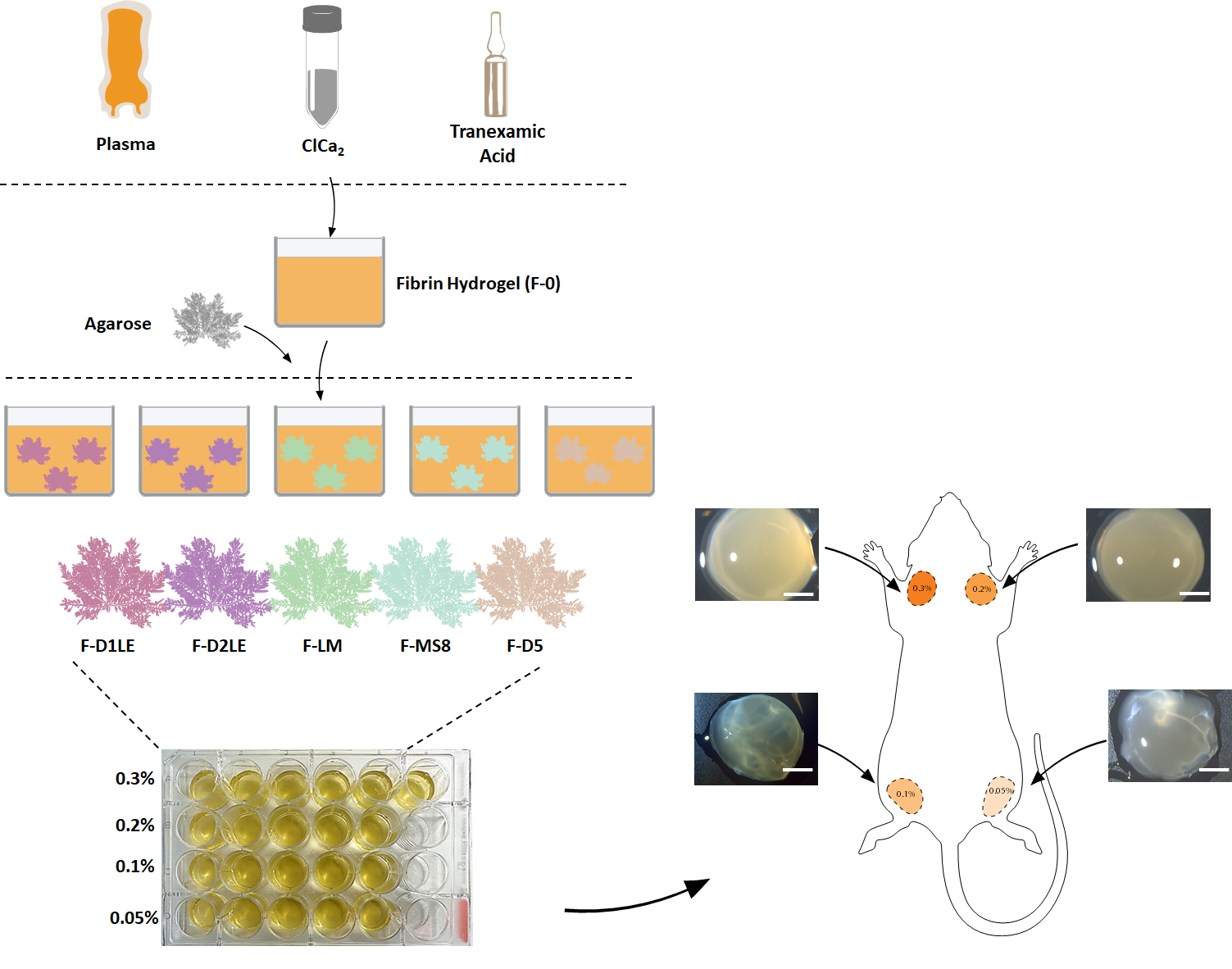

Supplement: Supplementary file 1 [file marinedrugs-21-00187-s001.zip › Supplementary Figure S1.tif]
